# Supplementary material for: STRIPAK Dependent and Independent Phosphorylation of the SIN Kinase DBF2 Controls Fruiting Body Development and Cytokinesis during Septation and Ascospore Formation in Sordaria macrospora
Source: J Fungi (Basel). 2024 Feb 26;10(3):177. doi: 10.3390/jof10030177 (PMC10971303; doi:10.3390/jof10030177)
Supplement: Supplementary file 1 [file jof-10-00177-s001.zip › jof-2868136-supplementary.pdf]

Supplementary Materials from Shariatnasery et al.

Table S1. *S. macrospora* strains used in this study.

Strain	Relevant genotype and phenotype	Source / Reference
R19027	Wild type, fertile	[1]Culture collection
S70823	Spore color mutant, fertile *fus1-1*	[1]Culture collection
S96888	Strain for homologous recombination, fertile Δku70::*nat*	[70]
Δdbf2	ASI from crossing of TMS756 and *fus1-1*, sterile *dbf2::hph*^{r} (Δdbf2)	This work
Δdbf2::OEdbf2	ASI, ectopic integration of *dbf2* into *Δdbf2* , fertile *Δdbf2::hphr::gpd(p)::dbf2::trpC(t)::nat*	This work
Δdbf2::NAdbf2	ASI, from ectopic integration of *dbf2* into TMS7569, fertile *Δdbf2::hphr::dbf2(p)::dbf2::trpC(t)::nat*	This work
Δdbf2::OEdbf2-gfp	ASI, ectopic integration of *gfp-dbf2* into TMS7569, fertile *Δdbf2::hphr::gpd(p)::gfp::dbf2::trpC(t)::nat*	This work
Δdbf2::OEdbf2-S104A	ASI, from ectopic integration of gfp-*dbf2*-S104A into TMS7569, fertile *Δdbf2::hphr::gpd(p)::gfp::dbf2S104A::trpC(t)::nat*	This work
Δdbf2::OEdbf2-S104E	ASI, from ectopic integration of gfp-*dbf2*-S104E into TMS7569, fertile *Δdbf2::hphr::gpd(p)::gfp::dbf2S104E::trpC(t)::nat*	This work
Δdbf2::OEdbf2-S502A	ASI, from ectopic integration of gfp-*dbf2*-S502A into TMS7569, sterile *Δdbf2::hphr::gpd(p)::gfp::dbf2S502A::trpC(t)::nat*	This work
Δdbf2::OEdbf2-S502E	ASI, from ectopic integration of gfp-*dbf2*-S502E into TMS7569, sterile *Δdbf2::hphr::gpd(p)::gfp::dbf2S502E::trpC(t)::nat*	This work

ASI: ascospore isolate, *nat*^{r}, nourseothricin resistance gene; *hph*^{r}, hygromycin B resistance gene, *gpd(p),* constitutive *gpd* promotor from *Aspergillus nidulans* [49], *trpC(t), trpC* terminator from *Aspergillus nidulans* [68], [1]General and Molecular Botany, Ruhr-University Bochum, 44780 Bochum, Germany

Table S2. Plasmids used in this study.

Plasmid	Relevant features	Reference
pDest-Amp	Destination vector for Golden Gate cloning; *bla* (Bsamut), *lacZ* gene with two internal *BsaI* sites *BsaI(4)* and *BsaI(7)*	[39]
pD-GG-KO-hph	*trpc(p)::hph* in pDrive; *bla, kan, lacZ* gene with two internal *BsaI* sites *BsaI(4)* and *BsaI(7)*	[39]
pDS23-pRSnat_eGFP_ITneu	*gpd*(*p*)::*gfp*::*trpC*(*t*), *trpC*(*p*)::*nat*, *URA3*, *bla*	Teichert (pers. communication)
pMSD16-dbf2	1.2 kb 5′ flank region and 1.2 kb 3′ flank region of *dbf2* with *trpC(p)::hph::bla* in pDest-Amp/ pD-GG-KO-hph	This work
pOE*dbf2*	*gpd(p)::dbf2::trpC(t)* in pDS23-pRSnat_eGFP_ITneu	This work
pNA*dbf2*	*dbf2(p)::dbf2::trpC(t)* in pDS23-pRSnat_eGFP_ITneu	This work
pNA*dbf2*-eGFP	*gpd(p)::gfp::dbf2::trpC(t)* in pDS23-pRSnat_eGFP_ITneu	This work
pOE*dbf2*-S104A	PMSC8-eGFP carrying *df2*$_{S104A}$ mutation	This work
pOE*dbf2*-S104E	PMSC8-eGFP carrying *dbf2*$_{S104E}$ mutation	This work
pOE*dbf2*-S502A	PMSC8-eGFP carrying *dbf2*$_{S502A}$ mutation	This work
pOE*dbf2*-S502E	PMSC8-eGFP carrying *dbf2*$_{S502E}$ mutation	This work

Table S3. Oligonucleotides used in this study.

Oligonucleotide	Sequence (5′-3′)	Specificity
hph1IT	atccgcctggacgactaaac	3′ *hph* forward
hph2-IT	ggctgtgtagaagtactcgc	5′ *hph* reverse
MS-dbf2-5-fw	ACGACTGGTCTCAAGTCcacccacttactgccactg	*dbf2* 5′ flank forward
MS-dbf2-5-fw	ACGACTGGTCTCAAGTCcacccacttactgccactg	*dbf2* 5′ flank reverse
MS-dbf2-3-fw	TCGTACGGTCTCGGTggcaacaccaacaggaggatg	*dbf2* 3′ flank forward
MS-dbf2-3-rv	ATCTCAGGTCTCCCGTAaggagaagttggctaggtatg	*dbf2* 3′ flank reverse
5230-dbf2-5-fw2	gaatggagctttctggagcg	upstream of *dbf2* 5′ flank forward
5230-df2-3-rv2	accaccagtccttccaaaac	downstream of *dbf2* 3′ flank reverse
1751-fw	gccatattttcctgctctcc	*gpd(p)* forward
MS-egfp-fw	ggtgaacttcaagatccg	*gfp* forward
1757-rev	agctgacatcgacaccaacg	*trpC(t)* reverse
DBF2_pNATIVE-(fw)	ctcatatgcttggccttgtcagctttgc	5' flank with pDS23 overlap
DBF2_pNATIVE-(rv)	taagatctgactgaaagctatgccag	3' flank with pDS23 overlap
MS-pura3-(fw)	ggaaggagcacagacttag	upstream of *pNative-dbf2* forward
MS-dbf2-48-HR(fw)	gactaacagctacagatctaagcttatgtcgagctacataacaaacttcttccccagcggc	5' flank with pDS23 overlap
MS-dbf2-48-HR(rv)	ttaagtggatccactagttctagatctacagcatcgtaccaaaagtgttgc	3' flank with pDS23 overlap
MS-dbf2-GFP-HR-(fw)	ctctcggcatggacgagctgtacaagtcgagctacataacaaacttcttc	5' flank with pDS23 overlap
MS-dbf2-GFP-HR-(rv)	gatttcagtaacgttaagtggactacagcatcgtaccaaaagtgttgc	3' flank with pDS23 overlap
Q5MSDBF2-104ser::Ala_F	CACACCCCTAgCCCCTGGAAAG	*dbf2* S104A
Q5MSDBF2-104ser::Ala_R	GCCACACTCTGGGGACGG	*dbf2* S104A
Q5MSDBF2-104ser::Glu_F	CACACCCCTAgagCCTGGAAAGAGCAACCTC	*dbf2* S104E
Q5MSDBF2-104ser::Glu _R	GCCACACTCTGGGGACGG	*dbf2* S104E

Q5MSDBF2-502ser::Ala_F	CTACGCCAAGgccATTGTTGGATC	*dbf2* S502A
Q5MSDBF2-502ser::Ala_R	TTGGTATCCCGATCACGC	*dbf2* S502A
Q5MSDBF2-502ser::Glu_F	CTACGCCAAGgaGATTGTTGGATCTCC	*dbf2* S502E
Q5MSDBF2-502ser::Glu_R	TTGGTATCCCGATCACGC	*dbf2* S502E

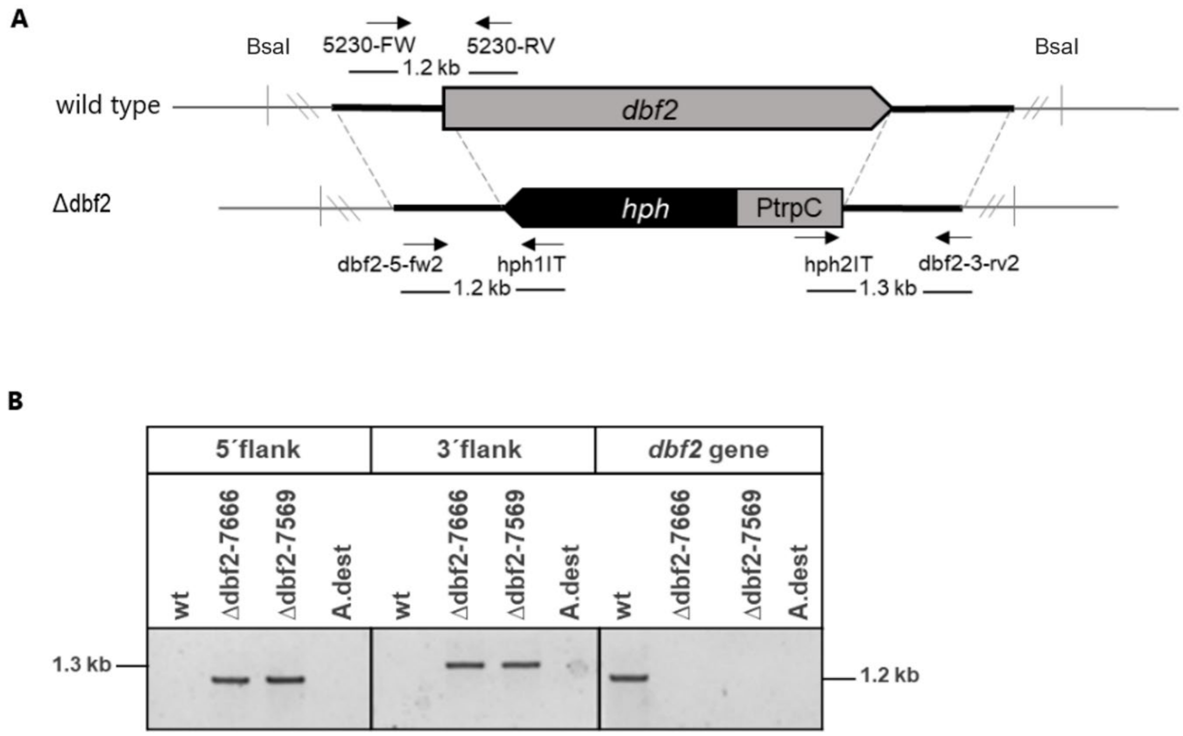

Figure S1. Generation and verification of Δdbf2 strains. (A) Genomic organization of the dbf2 locus in the wild type and Δdbf2 deletion strain [70]. In Δdbf2, the *dbf2* gene is substituted by the hygromycin B phosphotransferase coding gene (*hph*) under control of the trpC promoter [71]. Arrows indicate oligonucleotides used for PCR amplifications. Thin black bars represent expected fragments in the PCR analysis. (B) PCR verification of homologous recombinant 5′ flank, 3′ flank and *dbf2* gene in the wild type (WT), and sterile mutants Δdbf2. Distilled water (A. dest.) was used as a PCR control.

	S104	S502
dbf2 wt	/cccctatcccctggaaag/<br>P L S P G K	/gccaagtcgattgttgga/<br>A L S I V G
dbf2 S::A	/cccctagcccctggaaag/<br>P L A P G K	/gccaaggccattgttgga/<br>A L A I V G
Dbf2 S::E	/cccctagagcctggaaag/<br>P L E P G K	/gccaaggagattgttgga/<br>A L E I V G

Figure S2. Functional analysis of *dbf2* was conducted using phospho-mimetic and phospho-deficient variants. The coding sequence of *dbf2* and its derived amino acid sequence were indicated with lowercase and capital letters. The triplets encoding the phosphorylated amino acids were highlighted in bold and grey. Single base pair substitutions and the resulting amino acid substitutions (S104A, S104E, S502A, and S502E) were indicated in red.
